# Mr. Stevenson on the Nature and Treatment of Amaurosis

**Published:** 1822-03-01

**Authors:** 


*On the Nature, Symptoms, and Treatment of the different Species of Amaurosis, or Gutta Serena; Illustrated by Cases.* By JOHN STEVENSON, Esq. Surgeon-Occulist and Aurist to His Royal Highness the Duke of York, and His Royal Highness Prince Leopold of Saxe Cobourg: Member of the Royal College of Surgeons, &c. &c. One vol. 8vo. pp. 277. London, 1821.

---

" But thou
" Revisit'st not these eyes—that find no dawn;
" So thick *a drop serene* hath quench'd their orbs,
" Or dim suffusion veil'd."—MILTON.

---

THE fate of Homer, and the plaint of our immortal bard, have, as it were, consecrated amaurosis, and rendered it an interesting, though melancholy subject of contemplation. It is often so insidious in its approach, as to give no warning till the patient unexpectedly finds one eye entirely deprived of sight. About ten years ago, an interesting young lady of our acquaintance, was standing on the Calton Hill, surveying the romantic scenery that surrounds that enchanting spot. She took a small telescope from the hand of a gentleman present, and shutting one eye, applied the instrument to the other, in order to view the opposite coast of Fyfe. She found herself, to her astonishment and dismay, in utter darkness. The right eye was completely amaurotic, while the left was perfectly sound. Occulists were applied to; but gradually the remaining eye lost its function, and the unfortunate lady is now blind, though in the prime of life!

The attention of Mr. Stevenson appears to have been drawn towards the subject of amaurosis, in 1804, by being called to an apoplectic patient, whose person was tall and thin, countenance pallid, and habits remarkably regular. Yet on dissection, besides extravasation of blood pressing on the tractus opticus, and causing blindness in the beginning of the paroxysm, the most decisive marks of vascular congestion were observed in the meninges of the brain. This case, our author remarks, served to shew "that *pressure* is the immediate cause of amaurosis." This is, perhaps, too general a conclusion. That pressure on the optic nerve will cause blindness, cannot, we believe, be doubted; but, that amaurosis is *always* caused by pressure, we can scarcely admit, nor do we suppose that Mr. Stevenson means to draw such a conclusion.

Besides an attempt to elucidate the subject of amaurosis, Mr. Stevenson hopes to prove, by an appeal to facts, as well as reason and analogy, that the disease under consideration admits, in various instances, "*when unaccompanied with morbid changes of structure in any part of the eye-ball*," of successful modes of treatment. This exception narrows the hope of cure, however, very considerably.

The work consists of four chapters—namely, on the definition and symptoms, the prognosis, exciting causes, and treatment. To these are added, an Appendix of Cases.

*Chap*. I. It is not very frequent that we find the etymology of a disease convey a just notion of its nature. Thus, the term amaurosis signifies merely dimness or obscurity of sight—and the term gutta serena was adopted, because the disease was supposed to arise from a drop of lymph, the pupil retaining its natural pellucidity. Mr. Stevensons's definition is—" that disease of the *eye* which consists in a diminution or total loss of sight, without any other visible imperfection in the organ, than a dilated and immoveable state of the pupil."* This definition, however, Mr. S. acknowledges, will not meet the varied symptoms of each particular case—an imperfection not confined to Mr. Stevenson's definition alone. Thus, Richter asserts, that a degree of *squinting* is the only symptom inseparable from gutta serena. Our author

---

* Richter asserts that, nothing positive can be drawn from the mobility or immobility of the iris, as sometimes the one, sometimes the other is the case. It is but right, however, to observe, that Mr. Stevenson confines amaurosis to an affection of the optic nerve and its expansion, solely. Whereas, he thinks, that the term in authors, has been extended, in some instances, to affections of the choroid, and even sclerotic coats, and of the internal parts of the eye-ball, and even of the brain.

allows that this symptom is generally present in patients with one eye amaurotic; but, in some of the worst species of the disease, in which both eyes are equally dark, the loss of control over the muscles allows them to act irregularly, occasioning that singular want of expression, vacant unmeaning stare, or rolling motion, as if the eyes were in fruitless search after the lost sight.

> " But thou
> " Revisit'st not these eyes that *roll in vain*,
> " To find thy piercing ray."—*Paradise Lost.*

Bearing in mind, however, that strabismus is frequently owing to defective action of one of the muscles of the eye—to sympathy with disordered states of the primæ viæ—to morbid habit, &c. still, when it co-exists with impaired or lost sight, and independent of any of the causes abovementioned, "it may be regarded as one of the least fallacious of the pathognomonic symptoms of amaurosis." It is a useful criterion in assisting our decision, where there is no other visible mark, and where the patient may have an interest in deceiving us.

At page 7, Mr. Stevenson considers dilatation and immobility of pupil, as furnishing only presumptive evidence of amaurosis; but, apart from collateral evidence, not to be admitted as undeniable proof of the presence of the disease. Our author has seen complete amaurosis, where the pupil continued to perform its function correctly. Amaurosis again, is not unfrequently accompanied with a fixed and contracted pupil—a species of disorder, Mr. S. thinks, most commonly the consequence of a violent internal ophthalmia, and usually combined with an angular, or irregular form of the pupillary border, in one or more points of its circumference, and with an opacity of the capsule of the crystalline lens, constituting the complicated adherent capsular cataract.

A paralysis of the ciliary nerves occasions a gaping and immoveable pupil, although the texture of the retina itself may not be affected.—On the contrary, the motions of the pupil may be active, notwithstanding the insensibility of the optic nerve, provided the ciliary nerves and the iris are free and uncompressed. From all this it is evident, that the conditions of the pupillary aperture, considered abstractedly, do not warrant any positive opinion relative to the precise character and nature of amaurosis. Auxiliary information, however, will be obtained by examining the pupil in a favourable light. When the disease is become inveterate, the pupil will rarely be found to exhibit that clear jetty brightness so constantly visible in a healthy state of the eye. It is more commonly of a dull horn-like blackness, an appearance which Mr. Stevenson thinks sufficient in itself to excite suspicions in the mind of the experienced observer—especially if accompanied by defective vision. Sometimes, Mr. S. observes, the colour of the pupil inclines to a sea-green tint—at others, there is a deep-seated, diffused turbidity, or muddy aspect of the pupil, which has caused it to be mistaken for cataract—an error that may be avoided by attention to the state of the pupil, (the mobility of which is not influenced by incipient cataract,) and to the relative situation of the opacity, as compared with the more forward position of the crystalline.

One of the earliest symptoms of cataract is, the perception of a *settled* mist before the eyes, at which period, dioptric glasses afford considerable relief—whilst in amaurosis they are generally useless.

" Another diagnostic symptom, strongly and very properly insisted on by writers on the subject, applies to the difference which the flame of a candle exhibits to persons respectively labouring under incipient Cataract, or Amaurosis. To the former it appears as if it were involved in a generally-diffused thin mist, or white cloud; but to the latter a halo, or the colours of the rainbow seem to encircle, or emanate from the mist. Again, the individual who is affected with the imperfect Amaurosis, usually experiences an occasional increase or diminution of his visual faculties, under different states of the circulation, as influenced by a full and stimulant meal, by which some find their sight improved, others greatly deteriorated. Enlivening or distressing mental emotions, and other physical causes which have a tendency to excite or depress the energy of the nervous system, have a correspondent effect in affording a temporary benefit, or in causing a diminution of vision, which do not occur in cases of incipient Cataract." 29.

Our author thinks that the form attended with a deep-seated muddy appearance of the eye, is caused by an alteration in the texture of the hyaloid membranous septa of the vitreous humour, constituting the disease called glaucoma. But one of the worst—indeed, altogether incurable species of gutta serena, is that which is marked by an opaque, dull whitish appearance at the fundus of the concave surface of the eye-ball, answering to the situation of the retina, in a morbid alteration of which it, indeed, consists. When this disease succeeds to deep-seated pain, it is probably the result of inflammation of the retina, which, like other transparent textures, becomes opake from inflammatory action, occasioned either by an effusion of lymph upon the interior of the choroid, or by an actual change of its proper structure.

" In these instances, the whole of the back part of its internal surface undergoes a similar change, the cloudiness appearing somewhat concave, the pupil more or less expanded, the iris with little, or not any motion, the pupillary border irregular, and the vision nearly, or altogether extinguished." 32.

Though sometimes produced by *acute* internal ophthalmia, this affection of the retina more frequently proceeds from insidious chronic disease. The following symptomatology we cannot abridge, and therefore we quote it.

"The symptoms accompanying this form of the disease are a varicose, or overloaded state of the superficial vessels of the eye, with a sense of tension and occasional uneasiness, rather than violent lancinating pains, indicating a congestion of the deep-seated vessels. The sclerotica, at the same time, frequently becomes thinner and semi-transparent, and admits of the reflection of the vascular texture of the subjacent choroid, which occasions a generally diffused livid, or purplish tint; or yielding, in certain points of its circumference, one or more staphylomatous protrusions are formed. The humours, losing their natural limpidity, assume a yellowish or muddy appearance, and are so greatly increased in bulk, as to press through the pupil, causing it to become widely dilated and immoveable, the thin irregular border of which appears to stretch around, and to embrace the bulging lens." 33.

It is hardly necessary to add that vision is wholly annihilated; though the patient is sometimes annoyed by the sensation of flashes of light—a proof that the sentient texture of the eye is involved in the general disorganization.

But instead of extensive changes in the tunics or humours of the eye, the whole sometimes appear sound, except on looking, by the aid of a good light, into the interior of the eye-ball, when an opaque white spot, or projecting chalk-like or pearly substance is visible at some part of the concave face of the retina, which is frequently covered with a network of coloured blood-vessels, derived from the arteria centralis retinæ. This disease has been termed *cancer of the eye*, and medullary fungus. It is not preceded by, nor, in its early stage, accompanied with pain or any of the usual phenomena of inflammation. At the commencement of this dreadful disease, and before the fungus has begun its luxurient growth, it appears in a detached molecula, at the fundus of the eye, resembling very much the soft medullary substance of the brain. In the progress of the malady all the structures of the eye become involved in an undistinguishable disorganized fungous mass. The constitutional derangements resulting from such a dreadful local irritation, may be easily imagined, and are well pourtrayed in the 36th page of the work before us.

"There is still another form of Amaurosis to be noticed, in which the bottom of the eye exhibits, in a strong light, a silvery or yellowish spot situated near the axis of vision. This appearance has been attributed to a circumscribed opacity of the retina, answering to the porus opticus. Others have supposed it to be the macula lutea of

Soemmering. It is however with more propriety ascribable to a diminished secretion of the black pigment, a defect which is sometimes experienced by persons advanced in life, or by those who have suffered under chronic wasting diseases, or choroideal inflammation. The eye, in this species of Amaurosis, ceasing to resemble, by its dark concavity, a camera obscura, like that of the Albino, is greatly distressed by strong light, and the partial illumination of objects, by exposure to which the sight is very much confused and temporarily impaired." P. 41.

The sight of persons labouring under amaurosis is very variously affected—some seeing only a part of the object before them, others having double or distorted vision, &c. To attempt to describe the various spectral illusions resulting from disordered states of the retina would be useless and unprofitable.

As the fixed muscæ are most commonly organic—so the volitantes acknowledge a functional origin, Mr. S. thinks—not unfrequently arising from sympathy of the retina with a deranged state of the chylopoietic viscera, and disappearing, of course, when the function of these organs is restored. The inability to adjust the focal power of the eye to *near* objects, without a sense of fatigue and weariness in the organ, so common with people as they advance in life, Mr. S. does not impute to a supposed weakness of the recti muscles—nor to a preternatural rigidity of them—nor to any change in the form of the cornea, or defective secretion of aqueous humour, but to "an actual diminution of nervous influence in the sentient texture of the eye itself." This doctrine, our author thinks, derives strong support from the fact that—"in such cases the lively movement of the iris, and the ability steadily to maintain its healthy action, is proportioned to the strength of the adjusting power of the eye."

Our author has known instances of amaurosis occurring to several children in the same family—thus evincing an hereditary tendency. The disease is most apt to occur in eyes of a blue and black colour, and where vision has been habitually weak and irritable. When one eye is amaurotic, the other is in danger.

Amaurosis sometimes makes its attack very suddenly. Mr. S. has met with several instances of patients going to bed apparently well, and awaking with more or less complete loss of vision in one or both eyes, the pupils being, at the same time, proportionally dilated, and unsusceptible of the impression of light. These cases were *functional*, and terminated favourably. It more generally happens that Amaurosis advances in a gradual and insidious manner, with such fluctuations in progress, as sometimes to inspire hope, and at others to induce despondency. Functional disease, if neglected, or too often repeated, may end in organic affection.

"Under ordinary circumstances," (our author observes,) "the patient first becomes sensible of a somewhat weakened sight, or rather of an interrupted state of vision, manifested by certain parts of small objects, as for example, the letters, or lines of a book, being at one time more distinctly visible than at another, the sight of which he alternately loses and regains by shutting or rubbing his eyes, or by moving his head in different directions. This symptom which, when it occurs, is the earliest intimation of the commencement of the disease, is to be regarded as a proof of the impaired sensibility of the optic nerve, and may perhaps be explained by supposing, that certain points of the retinal expansion, in consequence of the general diminution of its energy, speedily lose their perceptive faculty. Hence it becomes requisite, for the continuance of the function of the organ, that new surfaces should be successively presented, which may not yet have been exhausted by the impression of external objects. Or, the stimulus of attentively observing a small object, inviting a further afflux of blood to certain parts of the vascular texture of the retina, already possibly in a preternaturally turgid condition, may induce an additional accumulation of fluids, a consequent pressure upon, and a corresponding torpor in, a defined portion of its medullary lamina." 65.

On other occasions the patient has a perception as if a spider's web, or thin gauze were interposed between eye and object—or he is annoyed by various imaginary appearances, as motes, threads, or other fantastic figures floating before him. The eye, at this period of the disease, begins to lose somewhat of its natural brilliancy, the iris, in most instances, becoming more and more dilated, sluggish, and less sensible to light.

"Should one eye only be affected, its iris will still act in accordance with that of the sound organ, provided both be allowed to remain open at the same time; but if the latter be closed, the pupil of the really diseased eye will be found to have lost its power of motion, on the admission of the usual degrees of light." 68.

A young practitioner, Mr. S. observes, may avail himself of this fact, in order to avoid the error of incautiously pronouncing both eyes to be equally perfect in sight. In the incipient stage of the imperfect amaurosis (contrary to what is the case in cataract) the patient generally sees best in a strong reflected light—at which period, convex glasses are sometimes found to assist his sight. But when the malady is fully formed, he derives no assistance from this kind of spectacles. In some cases, however, strong light occasions distress to the organ of vision. Here there is reason to suspect that inflammation has pre-existed, and left the eye in a preternaturally susceptible state—or that morbid action is going on in the retina itself, or some of the adjoining textures.

*Chap.* II. *Prognosis.* This, as in most other diseases, must be founded on the peculiar nature of the existing causes. If the disease depend on organic lesion of the seat of vision, it may, of course, be considered incurable.

"When the sight is wholly extinguished, and the iris is immovably dilated, or preternaturally contracted, the effect of local or cerebral disorganization, symptoms which have been preceded by, or are accompanied with, violent pain of the cranium, the eye, or the forehead, the consequence of apoplexy, syphilis, blows upon the head, or eye-ball, or of obstinate internal ophthalmia; these, and other causes of a similar tendency, produce modifications of Amaurosis, or varied forms of the same disease, which may justly be pronounced, in almost every instance, absolutely incurable." 73.

On the other hand, those cases of imperfect amaurosis, which have not been ushered in by intense and long-continued cephalalgia, ophthalmodynia, or sense of great constriction of the eye-ball, and in which there are still some remains of sight, either laterally or in the axis of vision, with preservation of natural black colour in the pupil, will frequently admit of relief. Those cases of a periodical character also, provided they have not been allowed to run on long uncontrolled, may be regarded as of a favourable nature, since they are almost always referable to sympathy of the eye with gastric, hepatic, or intestinal irritation—or else they are symptomatic of hysteria, chlorosis, gout, &c. in which cases, the removal of the primary disease restores the suspended power of vision.

*Chap.* III. *Exciting Causes.* We shall pass over some introductory observations, physiological and pathological. Our readers are aware that, in the first number of this series, we endeavoured to shew that *pressure* on the encephalic mass, whether the blood was in or out of its proper vessels, was the great and almost universal cause of apoplexy. Mr. Stevenson has applied this doctrine to the etiology of amaurosis, and endeavours to prove, as far as proof can be obtained in these cases, that *pressure* on some portion of the optic nerve, whether thalamus, tractus, or retinal expansion, is the efficient cause of amaurosis in the great majority of cases. At the same time he does not carry the principle too far, nor deny that other causes, and even diametrically opposite ones to pressure, should occasionally produce the disease. He is ready to admit that, in a few cases, it results from inanition, after great loss of blood, or from mere debility, after protracted and exhausting diseases. To these may be added the operation of narcotics and sympathy with other organs.

"With these reservations, says Mr. S. the doctrine is as universally applicable as it is generally true."

Can it be doubted, he adds, that *pressure* acting upon the visual nerve, in any part of its course or expansion, is not invariably productive of greater or less derangement, or the total annihilation of its function, according to the degree of force with which that pressure has been applied? Here our author refers to the sepulchretum and other sources, for examples of pressure from tumours, &c. It must be confessed, however, that in some who have died amaurotic, no perceptible change could be discovered in the structure of the optic apparatus after death. The late Mr. Ware supposed that, in these cases, there was pressure on the nerve from dilatation of the anterior portion of the circulus arteriosus. But this was only conjecture. Mr. Stevenson is more inclined to attribute the disease to vascular congestion of the capillary vessels of the retina itself, the immediate seat of vision.

"It is, I may repeat, exceedingly probable from analogy, supported by well ascertained facts, that the greater part of the various and anomalous affections of the retina, characterized by impaired or depraved sight, without organic lesion, are the result of particular changes in its vascular structure, subsequently extending to and affecting its medullary lamina, although we are not, in the present state of our knowledge, able to point out the exact nature of those respective changes upon which the morbid symptoms depend." 108.

For the arguments and illustrations of this subject, we must refer to the work itself, from page 109 to page 121.

The *external* local causes of amaurosis demand much consideration. A large class of diseases included in the term "weakness of sight,"* are referable, our author thinks, to an affection of the retina and choroid coat, produced by intense application of the organ to the inspection of minute and dazzling objects.

"Hence the frequency of weakness of sight amongst silk stocking weavers, milliners, embroiderers, and other mechanics and artists, whose occupations oblige them to exercise their visual organ with too little intermission and variety, in looking intently at their delicate

---

* On weakness of sight, 3d ed. chap. i.

light coloured and highly illumined manipulations. Persons addicted to read, write, or perform much fine needle-work by the aid of candles, and what is much worse, by the brilliant and artificial light of lamps, rarely fail, if their organ of vision be constitutionally feeble, to discover, sooner or later, a greater or less decay of sight" P. 123.

Our author, in a former treatise, has endeavoured to shew that the defective vision, in the above cases, does not depend on primary local debility in the optic nerve or expansion, but that it acknowledges, as its proximate cause, an inflammatory, or at least a preternaturally turgid condition of the blood-vessels of the retina and choroid coat.

Although light be the natural stimulus of the eye, yet, like all other stimulants, if inordinately applied, it will occasion local plethora, and morbid sensibility. The relief, in such cases, of the more urgent symptoms, by local and general depletion, is a strong confirmation of the doctrine.

While a moderate degree of congestion in the ophthalmic vessels determines what our author denominates weakness of sight, a higher degree of vascular excitement announces the presence of *acute* retinal inflammation, characterized by greater intolerantia lucis, more distressing and deep-seated pain, referred to the fundus of the orbit, and darting frequently to the temples, occiput, and through different parts of the head; contracted pupils; ocular spectra, in the shape of drops of blood; sparks of fire, or meteoric flashes; disproportionate external redness; constitutional derangement, as quick hard pulse, and obstinate watchfulness.

If these symptoms be not speedily combated, the pain and sensibility to light diminish, the pupil begins to lose its preternatural contraction, and the sight becomes more and more imperfect, until, in a short time, the iris is fully and immoveably dilated, unless fixed by adhesive inflammation, the sense of sight being generally irreparably destroyed. The following is a summary of our author's pathology of amaurosis.

"The above observations warrant, I conceive, the following corollaries, viz. that a certain degree of fulness of the blood-vessels of the retina is essential to the healthful state of the organ; that an increased plethora, or inflammatory condition of them deranges their function, and produces the disease termed "Weakness of Sight;" that more active symptoms of the same nature, constitute acute inflammation of the retina, or deep-seated ophthalmia. In the chronic state of the disease, the increased bulk produced by the distention of the vessels of the lamina of the retina, occasions pressure upon its medullary texture, and deprives it of its natural sensibility, or in other words, induces paralysis; amaurosis, as already remarked, being a common result of internal inflammation of the eye. Between these different degrees of a plethoric, and truly inflammatory condition of the immediate seat of vision, there may be an almost infinite number of shades and gradations, occasioning that variety, and anomalous character in the symptoms, which are observable in the different affections of the retina."* 133.

Another obscure but important class of exciting causes of amaurosis must be sought in irritations or derangements of the abdominal viscera. In our present state of knowledge we cannot account for the various sympathies of distant organs, and therefore we shall not spend time on the *quomodo*, while in search of the *quo*. In this class, Mr. Stevenson thinks, we cannot regard the amaurotic symptoms thus produced, as indications of an organic or structural, but simply of a functional derangement or torpor, constituting the symptomatic species, tractable, in general, if taken early. It is almost unnecessary to say that, in these cases, the origin, or visceral source of the disease, must be sought, otherwise little impression will be made by remedies directed to the *apparent* seat of the morbid affection.

Mr. Stevenson next adverts to certain narcotic and poisonous substances which, when applied locally to the eye, or sympathetically through the medium of the stomach, are supposed to produce gutta serena. He thinks their operation, especially when taken into the stomach, is to produce an additional afflux of blood to the brain, and thus to oppress and paralyse the medullary substance of the retina. This, he thinks, is manifest from the fact that the impaired sight, consequent upon the use of certain narcotics, has been relieved,

* Lullier-Winslow, who has written a very short article on Amaurosis in the first volume of the *Dictionnaire des Sciences Medicales*, divides the disease, as far as etiology is concerned, into three species, which division we shall give in his own words, as he has somewhat anticipated Mr. Stevenson relative to local plethora being very generally concerned in the production of amaurosis.

"1mo. Amauroses idiopathiques *par plethore ou faiblesse locales*, par lesions organiques des nerfs de l'œil, on des parties qui en sont voisines.

"2ndo. Amauroses sympathiques *par plethore*, ou par faiblesse generales, par lesion des fonctions d'un organe ou d'un viscere, par lesion de la sensibilité générale.

"3tio. Amauroses metastatiques."

Lullier-Winslow observes that idiopathic amaurosis may be produced by all or any of those causes which occasion much irritation in the globe of the eye, and consequently *a plethora of the blood-vessels there*—such as blows, intense and deep-seated ophthalmia—too much reading where the type is very small—the reflection of the sun from sands and snows in certain countries—long-continued microscopic observations—reading by too obscure a light, which strains the eye—blows and injuries of the head, &c. *Ed.*

and sometimes wholly cured, by topical depletion, rubefacients, and other modes of counter-irritation.

Our author concludes the subject of etiology, by briefly considering that species of amaurosis, supposed to arise from causes which operate indirectly upon the nerve of vision, by inducing a sudden and very considerable reduction of constitutional vigour, or whose debilitating influence is confined to the eye itself. We all know, that extremes approximate, and that inanition will produce effects very similar to congestion. It is well-known, also, that derangements of balance in the circulation, are more liable to take place in the weakly, than in the vigorous constitution; for nothing is more common, than to see a great local congestion, or even inflammation, combined with general debility. It is a curious fact that, in numerous experiments on animals bled to death, there was always found local congestion, inflammation, or effusion, under the membranes of the brain. Amaurosis, however, from want of sufficient fulness in the blood-vessels, is exceedingly rare, when compared with those cases, which are the offspring of a plethoric condition of the vessels of the eye. The continental writers consider gutta serena, as frequently produced by the translation of gout, rheumatism, syphilis, porrigo, scabies, &c. upon the nerve of vision, and we have little doubt of the fact, however sceptical most of our brethren in this country may be on the subject. At the same time, we do not consider the case as a mere translation of *matter*, but a vicarious morbid *action* sometimes set up in the organ of sight, in consequence of the abovementioned diseases having been injudiciously disturbed in their course.

*Chap.* IV. *Treatment.* Mr. Stevenson observes that, writers have, for the most part, contented themselves with describing amaurosis as a morbid affection of organ, consisting in a weakness, debility, loss of tone, or exhaustion of the optic nerve, or retina. And this naturally led to an indiscriminate employment of powerful, general, and topical stimulants, without regard to the species, exciting causes, or existing phenomena of the disease. Mr. Stevenson thinks that, error has been committed in the treatment of amaurosis, in consequence, probably, of practitioners not being aware that, a congestion, or even inflamed state of the retina, is not indicated by the same train of symptoms, which bespeak a similar condition of the more external textures of the eye. These states are often to be inferred *solely* from an alteration, or suspension of function in the organ.

We have found some difficulty in following our author through this division of the work, in consequence of his so frequently lapsing backwards into the same train of pathological discussions, pursued in a preceding chapter, and here reiterated, though not exactly totidem verbis.

Beer suggests a division of amaurosis into two classes—one attended with diminished irritability of the whole eye, the patient seeking a strong and brilliant light—the other characterized by tenderness and irritability of the organ, the patient being averse to bright and vivid light.

"A more practical and useful division of the subject is, I conceive, into *sthenic* or *acute*, and into *asthenic* or *chronic* Amaurosis, the former being the product of high arterial action either in the retina itself, or in the adjoining parts of the eye, in habits constitutionally strong and vigorous; the latter, the offspring of local congestion, from a relaxed and debilitated state of the system at large, and of the part affected in particular." 189.

The efficacy of emetics in amaurotic cases, arising from derangement of the primæ viæ, but immediately dependent on local plethora, is discussed by our author, amidst some pathological speculations, for which we refer to the work. This efficacy he attributes, not so much to cleansing the stomach, as to restoring the balance of the circulation, and thus counteracting local congestion, or inflammation. Purgatives, since the days of Hippocrates, have been highly esteemed in ophthalmic inflammations, and, on the same principle, they must be beneficial, our author remarks, in amaurosis dependent on vascular excitement, or oppression of the retina. The following, contains the text of Mr. Stevenson's therapeutics in this disease.

"From what has been advanced, the indications of cure must consist in relieving the retina from vascular excitement or oppression, the effect of local inflammation, or simple congestion, by general or topical depletion, proportioned to the urgency of symptoms and the character of the disease, and by other means calculated to derive from the part affected, by removing obstructions and equalizing the circulation, and finally, by restoring the tone of the vessels and the impaired energy of the nervous texture of the eye." 203.

In robust habits, and where the causes have been such, as indicate the acute form of the disorder, both topical and general bleeding must be resorted to with promptitude and decision, in order to arrest the disorganizing process.

"And it should not be concealed, that a much larger quantity of blood must be abstracted in the primary retinal, or choroidæal inflammation, than in common cases of ophthalmia, in order to make such an impression on the morbid state of the deep-seated vessels, as to check the inflammatory action of the larger arterial branches, and enable the overcharged capillaries to recover their power of healthy contraction." 204.

Mr. Stevenson recommends drawing blood from, and afterwards dividing, the temporal artery, (successfully practised, in cases of iritis, by Mr. Saunders,) as the most efficient method of subduing the violent action of the ophthalmic arteries. He avers that, he has seen symptoms threatening apoplexy, suddenly arrested by compression of the carotid arteries, when the pulsation in these vessels has appeared unusually strong, and the extremities felt colder than usual. Second, in point of efficacy, to arteriotomy, is opening the jugular vein, in our author's experience. If these rather painful processes will not be submitted to, cupping-glasses should be applied to the temples—or if this be objected to, blood must be drawn, in quantity proportioned to the urgency of the symptoms, from the arm, till syncope be induced.

"It is owing to depletion not having been carried far enough, and at a sufficiently early period of the disease, that I am disposed to ascribe the generally unfortunate termination of the *acute* species of amaurosis. Bleeding in some form, I repeat, is to be esteemed our sheet-anchor in these very formidable cases, and must be repeated at short intervals, until the violence of the symptoms shall have been subdued." 206.

As auxiliary means, the bowels must be acted on by calomel and jalap, followed by saline antimonials, so as to keep up a slight nauseating and purgative effect. To these means may be added, digitalis, topical applications of cold to the temples and forehead. The necessary regimen is sufficiently obvious.

The more urgent symptoms of the acute stage having subsided, the remaining congestive state of the vessels may be confided to leeches and blisters.

Mercury, Mr. S. observes, may be exhibited advantageously, during the active state of the disease, "so as to produce an early constitutional effect, for it will tend, not only to alter the morbid action of the vessels, but will serve likewise, after the inflammatory symptoms have subsided, to promote the absorption of any lymph that may have been effused during the preceding violent vascular excitement." P. 208.

"Should the pupil, as very commonly results from choroidæal inflammation extending to the iris, shew a disposition to contract, or have actually formed an adhesion with the capsule of the lens, the application to the eye of a filtered solution of equal parts of the extracts of belladonna and stramonium must, on no account be omitted; or a liniment of the former, with ungt. hydrarg. fort. united together by means of the yolk of egg, be freely rubbed upon the superior palpebra and eyebrow, with a view to prevent a synizesis, or permanent contraction, or obliteration of the pupil. Whilst it is of importance to exclude strong light, every kind of compress or bandage to the eye itself should be studiously withheld; as they never fail to exasperate the symptoms. And with regard to local applications, it is very doubtful whether they afford any real benefit in this stage of the disease: at all events stimulants are decidedly improper." 209.

The judicious practitioner will, of course, endeavour to ascertain the cause of the disease, and counteract that cause, if possible. Thus, for instance, should the amaurosis have succeeded to the suppression or disappearance of some habitual discharge, or cutaneous eruption—the sudden abatement of gouty or rheumatic inflammation, or the cessation of gonorrhœal inflammation, the measures to be pursued, will be sufficiently obvious.

In the asthenic or chronic form of amaurosis, the treatment will be considerably modified. Local depletion, in this case, is simply to relieve topical congestion—not to subdue arterial excitement. Blisters and rubefacients to the neighbourhood of the orbit, are useful counter-irritants. With the same view, errhines, as suggested by some of the ancients, and by the late Mr. Ware, will lessen local congestion by the discharge which they elicit from the mucous membrane of the nose, and by the irritation they excite in the immediate vicinity of the affected organ.

"Even the external application of stimulants to the eye itself which tend to promote an increased secretion of tears, such as the vin. opii, infusion of capsicum, ungt. hydrarg. nitrat. and the vapour of aq. ammon. pur. admitted to the cornea, are remedies which will be found to co-operate with bleeding and purging in fulfilling the general indications of cure." 215.

The continental writers recommend exposure of the eye to the full glare of a meridian sun, as a very powerful and useful stimulus. Our author has not experienced the effect of this in his own practice, but has learnt an instance of its success from a patient.

"I must now introduce to the notice of my readers a mechanical remedy which has not, I suspect, been adopted for the cure of Amaurosis: namely, *dry-cupping* applied to the ball of the eye, and its appendages. By carefully fixing a well-adapted strong glass fitted with an exhausting syringe upon the edges of the orbit, the instrument may be made capable of exerting a more or less powerful influence upon the organ of vision, in proportion to the extent to which the atmospheric air contained in the cupping-glass is exhausted. The effect of its application is to occasion a great redness and tumefaction of the eye-lids, an immediate distention of the vessels of the conjunctiva, and a bulging forward, or protrusion of the whole globe of the eye; the obvious tendency of which must be to relieve the deep-seated vessels by attracting the blood to the superficial order, and thus to produce a manifest and rapid alteration in the whole circulating system of the organ. I am informed by the gentleman who first named the remedy to me, that in one instance it was had recourse to with the happiest success, the patient being perfectly restored to sight, although a variety of means had been previously adopted without the smallest perceptible benefit." 217.

A pauper, twelve months deprived of sight by an attack of amaurosis, lately applied to our author. The patient complained of a sense of uneasiness in the eye and around the orbit, vision being completely extinguished, and the pupil remaining fully and immoveably dilated, when exposed to the strongest light. Immediately after the apparatus was removed, and when the organ exhibited the appearances above described, the patient exclaimed that he could now see the operator's fingers moving. The pupil instantly recovered its power of contraction, and the pain wholly subsided. Mr. Stevenson does not state the result of the case, but recommends further trials of the dry-cupping to his brethren.

As to electricity, our author's experience coincides with that of many distinguished practitioners, "not only as regards its general inutility, but as respects also its occasional injurious effects." If indeed the majority of amaurotic affections depend on an inflammatory or congestive state of the deep-seated vessels, we can expect little good but much harm from electricity. Even in those cases where the complaint may be considered to be a withering or decay of the optic nerve, what restorative influence can we expect from electricity?

Mercury, at first view, Mr. Stevenson observes, might not seem an appropriate remedy in the *chronic* form of amaurosis. But as the disease is frequently connected with more or less visceral obstruction, the primary source of the congestive state of the vascular apparatus of the brain and retina;—

"The utility of mercury in such cases may be ascribed, partly to its tendency to remove obstructions in the hepatic system, the frequent cause of cerebral congestion; and partly to its effect of increasing the action not only of the arterial capillaries, but also of the absorbents; a property evinced by the disappearance of tumours and extravasations under its use, and by the fact, that secondary symptoms of lues venerea are apt to supervene from the slight and insufficient administration of that remedy serving to promote the absorption of the virus, in cases of syphilitic chancres, without subsequently destroying the constitutional morbid action. By virtue of its general stimulant quality, the various functions of life are made to proceed with increased force and activity. From hence arises its efficacy in diseases accompanied with congestion from venous remora, which it overcomes, by calling into general action the vascular and absorbent systems, and restoring the lost balance of the circulation." P. 226.

If the disease arise from, or be connected with, great constitutional exhaustion, the consequence of profuse uterine or other discharges, chalybeates will be advantageously combined with other tonic remedies.

Mr. Stevenson takes occasion to reprehend Mr. Travers' sweeping proscription and even ridicule of all *local* remedies in amaurosis. "The faith yielded to such applications, says Mr. Travers, is a relic of the not very remote superstition which ascribed miraculous powers to the hand of a living king, or a dead culprit."* Mr. S. asks if the instances of amaurotic blindness which the late Mr. Ware and others assert, with apparent fidelity, to have been cured by mercurial snuff, are to be deemed mere delusions and unworthy of public credit? Mr. S. pertinently enough demands why, in cases of this description, are *blisters* declared by Mr. Travers to be a remedy of great value, "in some as temporary irritants only—in others, as irritants and drains?" Is there not, says Mr. S. a close analogy between the operation of epispastics, and errhines which he has represented to be altogether useless? Surgeons, we see, will disagree sometimes as well as doctors.

Mr. Stevenson's work is terminated by an appendix of cases, which we have not space to give any account of in this article.

Upon the whole, as far as experience in a general way enables us to judge, the work before us is characterized by rational doctrines, and energetic practice. The author has had ample field for observation, and has communicated the result, with candour and freedom, to his brethren at large, and without any attempt to enhance individual reputation by pretensions to peculiar or infallible modes of treatment. Some strictures might be made by hypercritics on a few portions of the work, in respect to composition and arrangement; but these flaws, if such there be, we leave to those whose taste leads them instinctively to prefer offal to wholesome food.

---

* Synopsis of Diseases of the Eye, p. 300.

